# Case Report: Double Micro-Guidewire Technique for Emergent Rescue of Proximal Stent Collapse During Recanalization of Nonacute Occlusion of Vertebral Artery

**DOI:** 10.3389/fneur.2021.671158

**Published:** 2021-09-03

**Authors:** Kun Zhang, Jin-Chao Xia, Hui-Li Gao, Bu-Lang Gao, Yong-Feng Wang, Zhao-Shuo Li, Tian-Xiao Li, Zi-Liang Wang

**Affiliations:** Henan Provincial Hospital of Cerebrovascular Diseases, Henan Provincial People's Hospital, Zhengzhou University, Zhengzhou, China

**Keywords:** cerebrovascular disease, atherosclerotic occlusion, stent collapse, complications, double micro-guidewires

## Abstract

Cerebral arteries are usually tortuous, and in the treatment of cerebrovascular diseases with stenting, a stent deployed may be collapsed at one end, leading to reduced blood flow and subsequent stent occlusion. Immediate rescuing measures should be implemented to prevent severe ischemic events. In this case report, we present a case with V4 segment occlusion of the right vertebral artery treated with endovascular stent angioplasty. An Enterprise stent deployed at the occlusion segment was collapsed at the proximal end after withdrawal of the delivery system. Immediate rescuing measures were taken by navigating a micro-guidewire through the lateral stent mesh at the proximal end into the stent lumen followed by advancing a second micro-guidewire right through the reopened proximal stent end into the stent lumen for deployment of a supporting balloon-expandable Apollo stent to prevent stent collapse. Follow-up digital subtraction angiography 6 months later demonstrated patent stents and unobstructed blood flow.

## Introduction

As a major health burden worldwide, strokes result in a high rate of morbidity and mortality (1, 2). Intracranial atherosclerosis accounts for ~5–10% of all strokes and transient ischemic attacks in the world (1), whereas in Asia, intracranial atherosclerotic stenosis is responsible for 33–37% of acute ischemic strokes (3). It is demonstrated that symptomatic nonacute large intracranial arterial atherosclerotic occlusion is an independent factor to predict poor outcomes, recurrent strokes, and major stroke problems across the world (4, 5). A large number of patients with chronic large intracranial artery occlusion continue to be symptomatic in spite of maximal medication treatment (6), and these symptomatic patients with chronic hemodynamic impair are at a high risk of future strokes (4). Even though no consensus exists on the best therapeutic approaches of nonacute large intracranial artery occlusion, endovascular recanalization with angioplasty alone or with stent angioplasty is feasible, safe, and efficacious in highly selected patients with improved clinical

outcomes after treatment (7–10). Based on computed tomography (CT) imaging, CT perfusion, magnetic resonance imaging (MRI), and clinical symptoms, the indications for endovascular recanalization are reported to be chronic occlusion of large intracranial arteries; recurrent ischemic neurological deficits (TIA or stroke); or progressive neurologic impairment symptoms, cerebral border zone infarction, and/or a decrease in cerebral blood flow and presence of a vascular bed at the distal end of the occlusion with the diameter of the occluded vessel exceeding 2 mm and the length being <15 mm. In the treatment of intracranial atherosclerotic stenoses, percutaneous angioplasty and stenting have significantly reduced the incidence of ischemic stroke, which has played an increasingly important role in neurology (11, 12). However, when a stent is deployed for the treatment of atherosclerotic stenoses or chronic occlusion of a tortuous artery, the stent struts at the proximal end may get in close contact or occlude after withdrawal of the inner micro-guidewire due to arterial tortuosity and stenoses, resulting in failure of endovascular devices to pass through the stent and even complete occlusion of the stented artery. In this case, acute revascularization of the occluded stent is necessary to guarantee unobstructed blood flow through the stent and prevent thrombosis or complete occlusion of the stented artery. We have encountered a case with occlusion of the V4 segment of the right vertebral artery, which was treated with deployment of a stent. The stent proximal struts got in close contact and occluded after the delivery system was withdrawn. Acute rescue management was successfully performed by using two micro-guidewires through the stent to deploy a support stent to open the collapsed stent struts.

## Case Report

**Figure 1:**
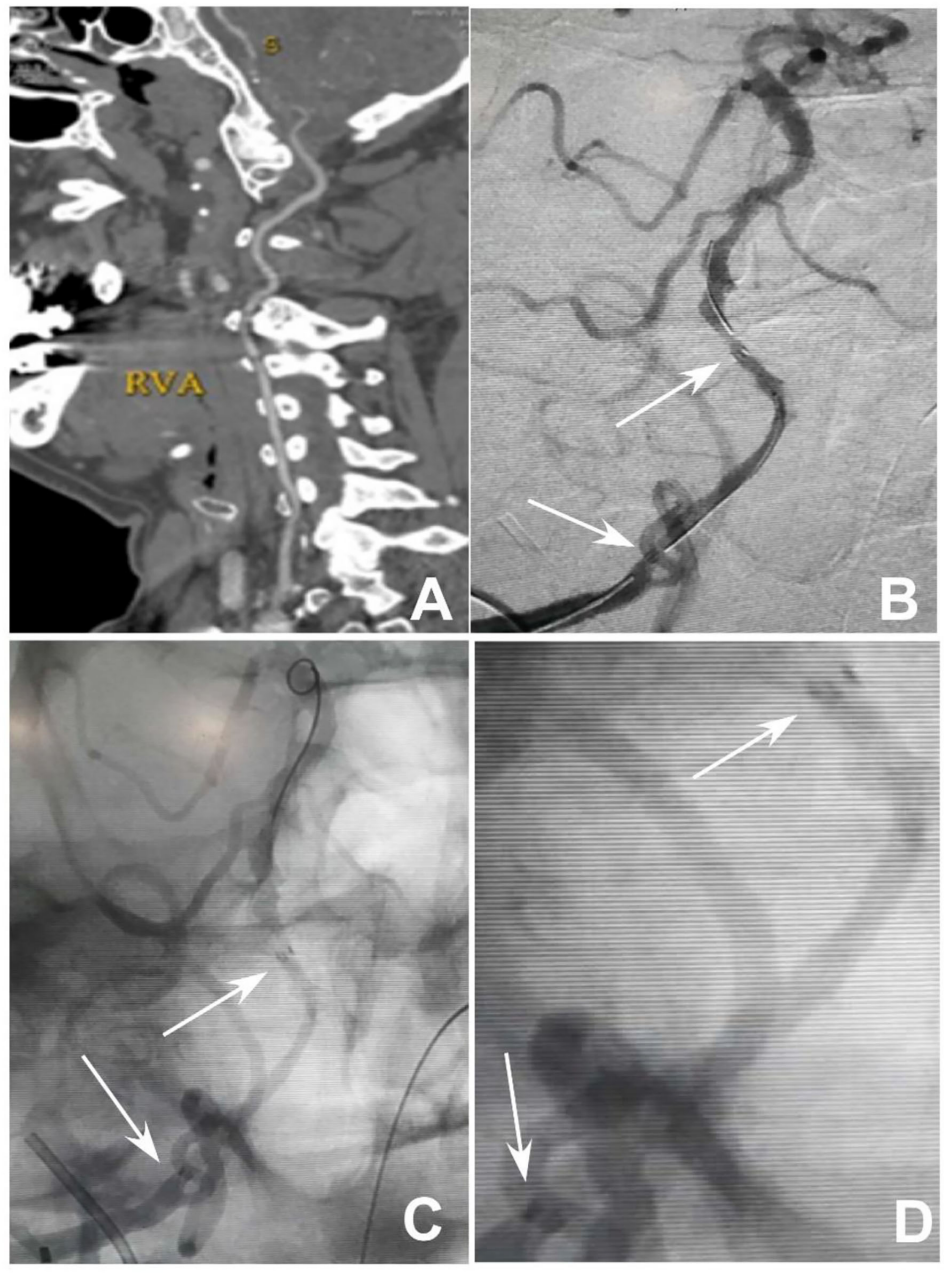
Endovascular treatment of V4 segment occlusion of the right vertebral artery. **(A)** Computed tomography angiography revealed occlusion of the V4 segment of the right vertebral artery without calcification of the occluded artery. **(B)** After balloon expansion of the occluded segment, an Enterprise stent was deployed at the occluded segment. **(C,D)** After deployment of the stent with the micro-guidewire still in the artery, the proximal and distal markers of the stent were spread out with patency of the stent. **(D)** is the local enlargement of **(C)** between the proximal and distal markers of the stent. Arrows indicate the proximal and distal markers of the stent.

**Figure 2:**
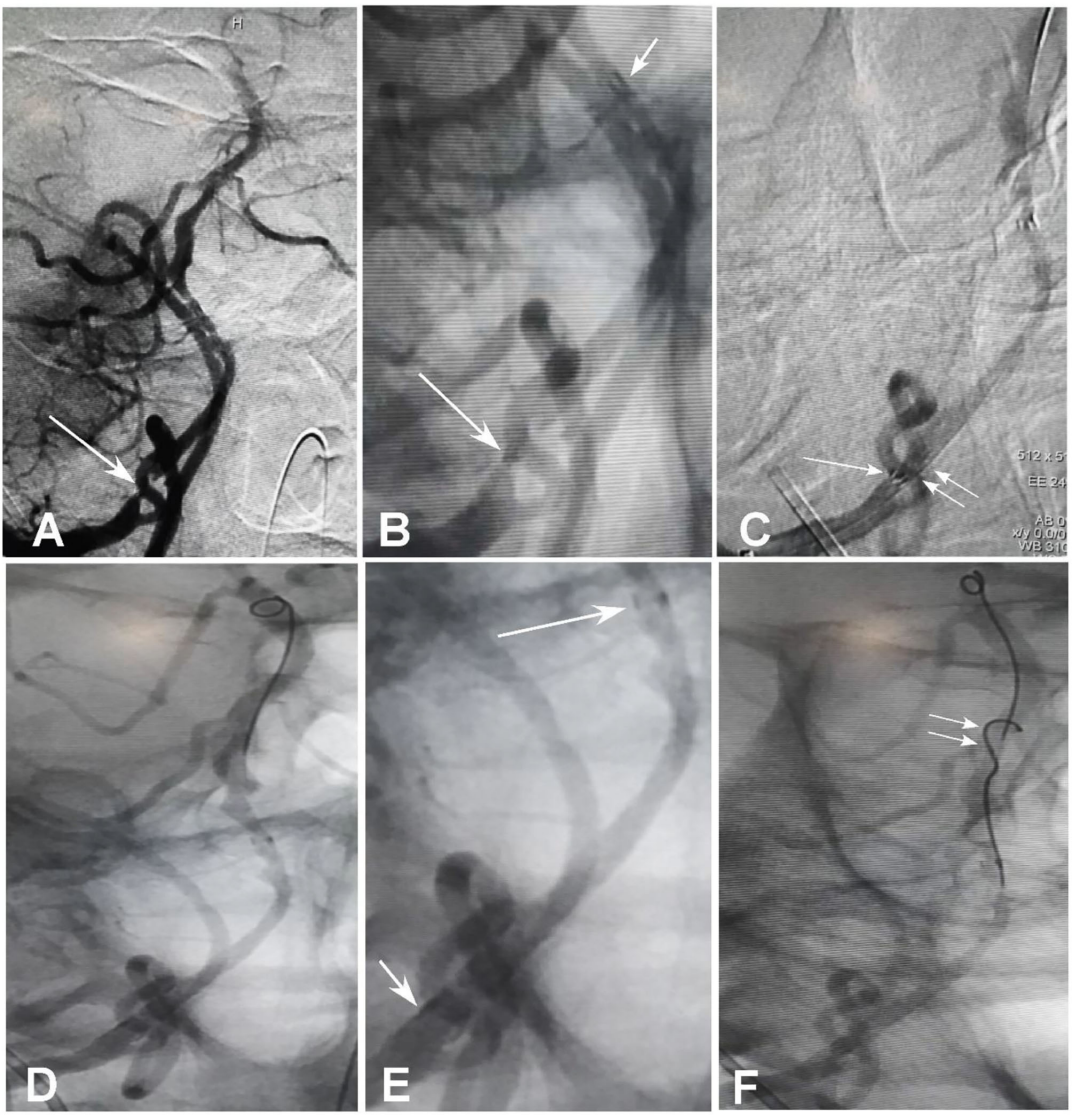
Collapse of the stent proximal end and management. **(A,B)** Withdrawal of the micro-guidewire and microcatheter led to collapse of the stent proximal end with the stent proximal markers in close contact, and the blood flow was reduced through the proximal end. The longer arrow indicates the proximal markers and the shorter arrow the distal markers. **(C)** A 300-cm micro-guidewire was navigated into the stent lumen through the proximal lateral stent mesh rather than through the proximal stent end. The longer arrow indicates the collapsed proximal markers of the stent, whereas the double arrows indicate the micro-guidewire through the stent lateral mesh. **(D,E)** After the micro-guidewire was sent to the distal segment of the basilar artery, the proximal stent end was opened with the proximal markers being spread out [**(E)**, shorter arrow]. The longer arrow indicates the distal stent markers. **(E)** is the local enlargement of **(D)** between the proximal and distal markers of stent. **(F)** A second 200-cm micro-guidewire was sent right through the opened proximal stent end into the stent lumen (double arrows).

**Figure 3:**
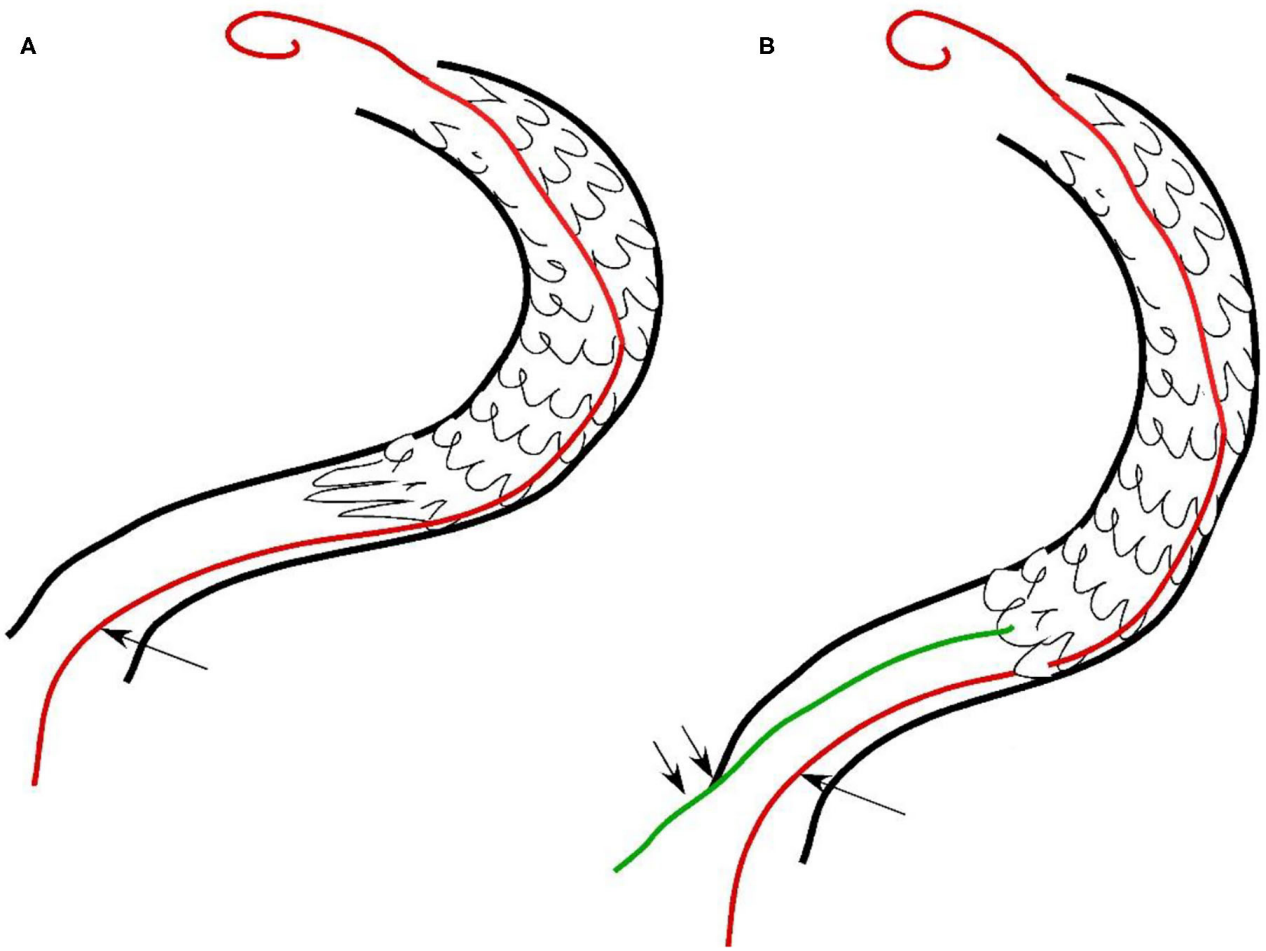
Hand sketch to show the deployment of two micro-guidewires. **(A)** The first micro-guidewire (longer arrow) was sent through the lateral stent mesh into the stent lumen after collapse of the stent proximal end. **(B)** After the artery was straightened by the first micro-guidewire with the stent proximal end being opened, the second micro-guidewire (double short arrows) was sent right through the opened proximal end for deployment of a supporting stent.

**Figure 4:**
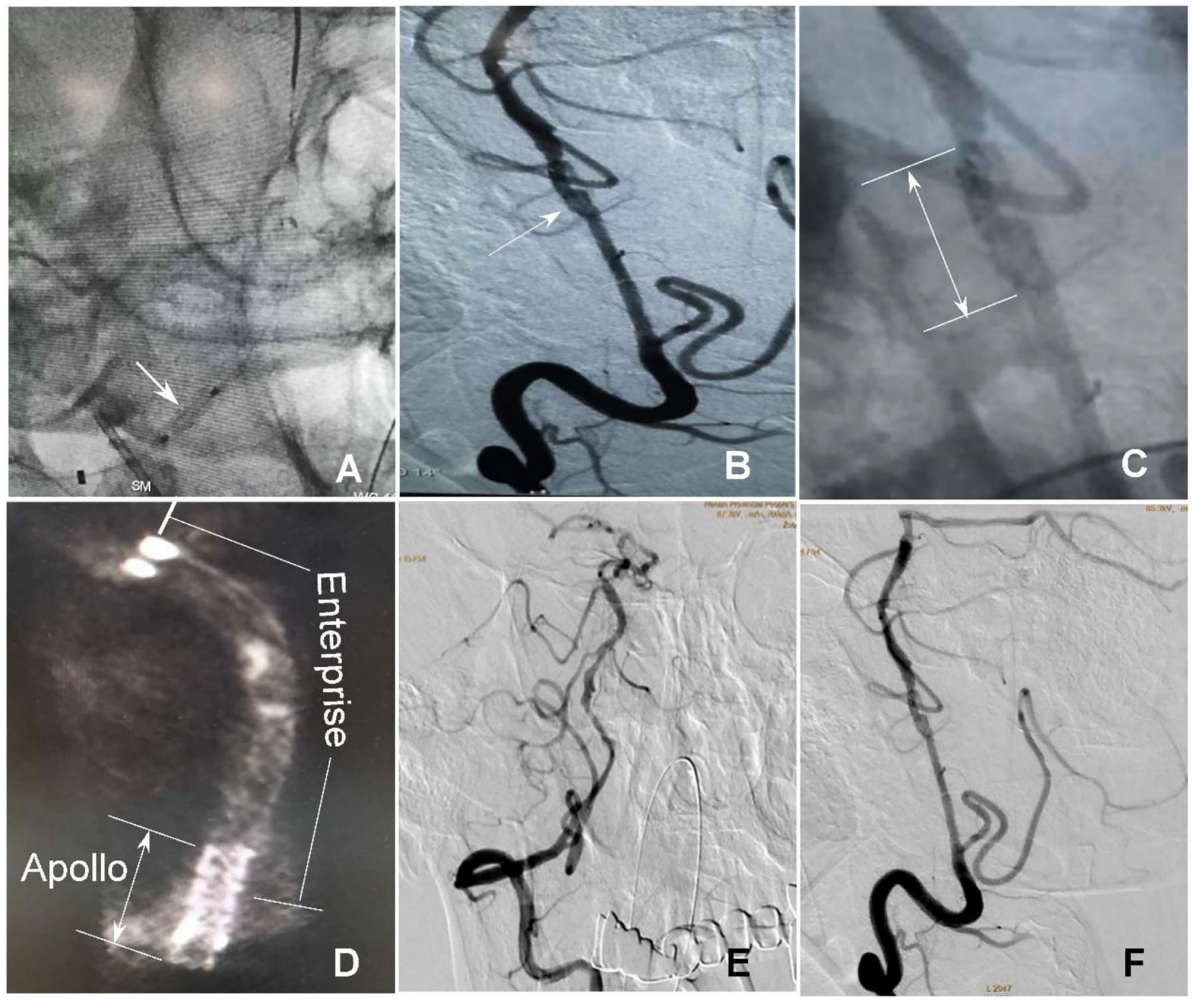
Rescue management of the collapsed stent end. **(A)** An Apollo stent (2.5 × 10 mm) was navigated along the 200-cm micro-guidewire to the proximal end of the Enterprise stent and expanded for deployment (arrow). **(B)** The Apollo stent was deployed to partially overlap with the Enterprise stent (arrow). **(C)** The arrow indicates the deployed Apollo stent. **(D)** DynaCT scan demonstrated partial overlap of the distal Enterprise stent with the proximal Apollo stent. **(E,F)** After stenting, both stents were well-patent with favorable forward flow.

**Figure 5:**
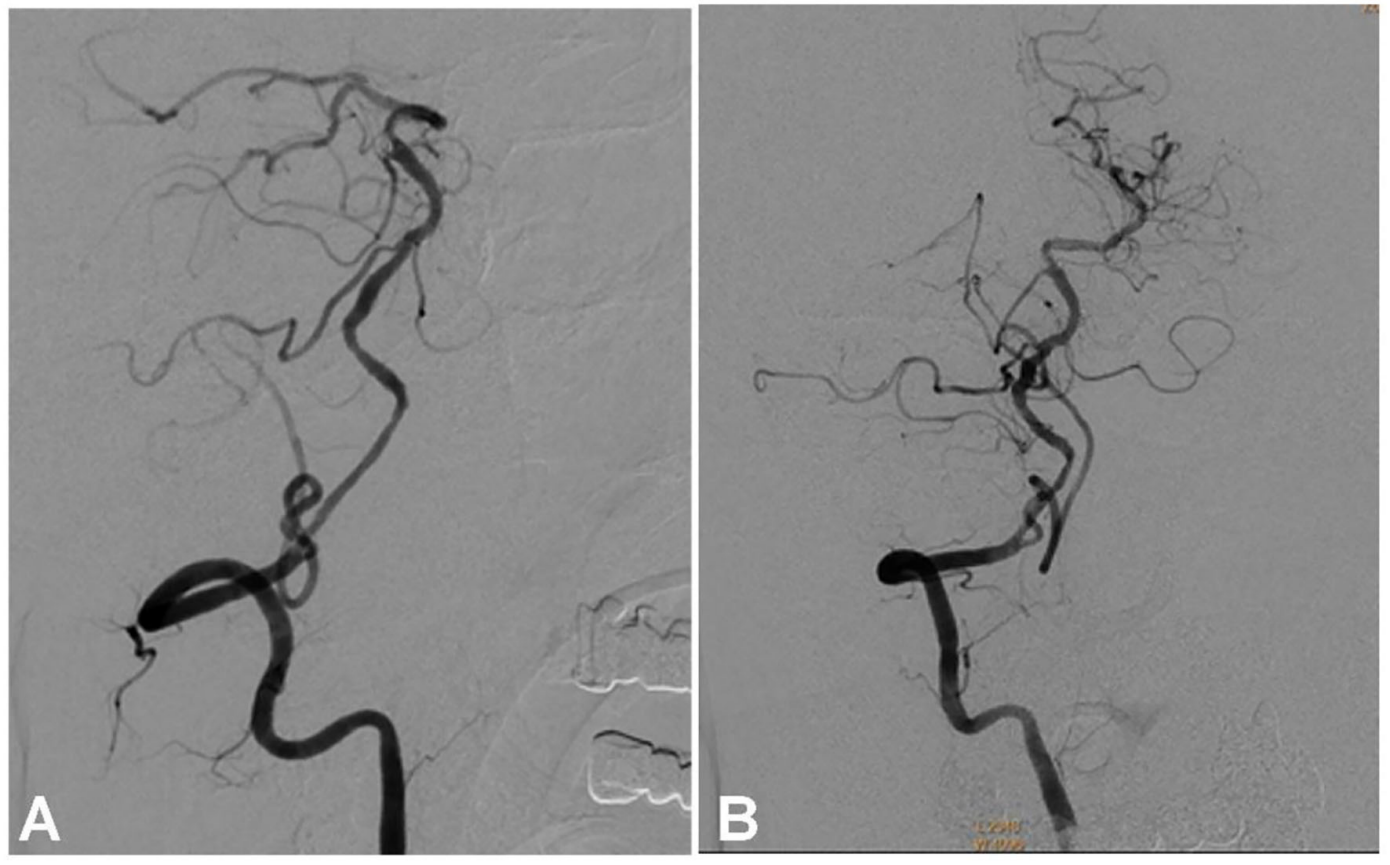
Follow-up angiography at 6 months after stenting demonstrates unobstructed blood flow through the stented vertebral artery **(A,B)** with no stenosis.

A male patient in his 50s had dizziness for 12 days that was not relieved after administration of medications. He had a history of smoking for 20 years with an average of six cigarettes per day and alcohol abuse for 30 years. Physical examination demonstrated blood pressure 123/78 mmHg, clear consciousness, slightly bad spirits, and no abnormality in the cranial nerve. Muscle strength and tension of limbs were normal. Auxiliary examination showed nothing abnormal in the electrocardiogram or laboratory tests (blood and urine routine, biochemical series, and coagulation items). MRI, MR angiography (MRA), and CT angiography (CTA) revealed occlusion of the right vertebral artery at the V4 segment (Figure 1A), which was confirmed by diagnostic digital subtraction angiography (DSA). The patient did not have a family history or genetic information of cerebral infarction or any previous endovascular treatment. Because medication did not perform well, the ischemic symptoms might be further aggravated, resulting in cerebral infarction and even death. To prevent aggravation of this condition, endovascular treatment was performed with written informed consent from the patient. The procedure was conducted under general anesthesia after administration of dual antiplatelet therapy with aspirin (100 mg/d) and clopidogrel (75 mg/d) for 3 days. The Seldinger technique was applied to gain percutaneous access to the femoral artery before insertion of a 6F introducer sheath, and heparin (70 U/kg) was administered intravenously to achieve an activated coagulation time of 150–200 s. DSA was performed to show the anatomy of the occluded artery, and a 300-cm Traxcess micro-guidewire (0.014 inch, Medtronic Inc., Minneapolis, MI, USA) was used to explore and navigate through the occluded segment before being put at the distal P1 segment of one posterior cerebral artery (PCA). Then, an Echelon 10 microcatheter (Medtronic) was sent along the micro-guidewire across the occluded segment to the same distal P1 segment of PCA, and gentle angiography through the microcatheter was performed to demonstrate the vascular structures. An angioplasty balloon catheter (Gateway, Boston Scientific, Natick, Massachusetts, USA) was advanced over the microwire, centered across the lesion, and inflated slowly for angioplasty (2.0 × 13 mm balloon) before an Enterprise stent (4.5 × 28 mm) was deployed at the occluded segment after accurate positioning (Figures 1B–D). At the time of stent deployment, the vertebral artery was straightened and smooth (Figure 1C), and the proximal end of the stent was wide open with the proximal markers of the stent being spread out (Figure 1D). Once the micro-guidewire and the conveying microcatheter were withdrawn, the stent proximal end was collapsed because of compression of the wall of the curved artery, which was exhibited by the closed proximal markers in close contact (Figures 2A,B). We tried to navigate a microcatheter (Excelsior SL-10, Stryker Neurovascular, Fremont, California, USA) into the proximal end of the stent, but this was unsuccessful. Then, the 300-cm micro-guidewire was navigated to the stent proximal end and passed through the proximal lateral mesh of the stent (Figure 2C). Once the micro-guidewire was sent to the distal segment of the basilar artery, the vertebral artery was straightened, and the stent proximal end was opened with the proximal markers being spread out again (Figures 2D,E). Then, a second 200-cm Synchro micro-guidewire was navigated right through the stent proximal end into the real lumen of the stent and sent to the distal segment of basilar artery (Figures 2F, 3A,B). After the 300-cm micro-guidewire was withdrawn, an Apollo 2.5 × 10 mm balloon-expanded stent (MicroPort Medical, Shanghai, China) was navigated along the Synchro micro-guidewire to overlap partially with the proximal segment of the Enterprise stent and was expanded with 4 atm pressure to support the proximal end of the first stent (Figures 4A–C). Angiography revealed good apposition of the two stents against the arterial wall with unobstructed blood flow through the stents and improved blood flow in the distal segment. Then, the micro-guidewire and microcatheter were withdrawn. Postoperative vertebral angiography showed complete recanalization of the occluded arterial segment (Figures 4B,C), and the Thrombolysis in Cerebral Infarction blood flow was grade 3. DynaCT scan revealed partially overlapped stents (Figure 4D). After stenting, both stents were well-patent with favorable forward flow (Figures 4E,F). At follow-up 6 months later, head CT showed no obvious abnormality, and physical examination revealed nothing abnormal in the cranial nerve. DSA demonstrated patent stents and unobstructed blood flow through the stents (Figure 5).

## Discussion

Atherosclerosis frequently involves the intracranial vertebral artery on bilateral sides (13, 14). Patients with atherosclerotic occlusion of both intracranial vertebral arteries often experience recurrent attacks of visual disturbance, dizziness, ataxia, or disability (14). Collateral circulation may provide baseline perfusion for some patients but fail to supply sufficient blood at times of increased oxygen demand, leading to lifestyle-limiting symptoms. The optimal treatment approaches for patients with intracranial vertebral artery occlusion is rarely analyzed. Given the high incidence and recurrent symptoms in spite of antiplatelet treatment, revascularization of the occluded intracranial vertebral artery using endovascular stenting and angioplasty has been attempted within the past decade and considered to be technically accessible because of fast development of endovascular management. However, the safety and efficacy of endovascular stenting and angioplasty still need further investigation (15–17). During the recanalization of intracranial vertebral artery occlusion, the major technical challenge is traversing the occlusion site with a guidewire even though other factors, such as the length and stage of the occlusion, may also affect the feasibility and outcome.

In this case report, occlusion of the right vertebral artery at the V4 segment was treated with endovascular stent angioplasty. During the intervention, the proximal end of the Enterprise stent was collapsed in the curved vertebral artery after withdrawal of the stent delivery system, resulting in reduced blood flow through the stented artery. The collapsed stent end was successfully rescued by deploying a balloon-expandable Apollo stent using the technique of double micro-guidewires. The deployment of the first micro-guidewire straightened the vertebral artery and reopened the stent proximal end, which facilitated passage of the second micro-guidewire through the opened proximal stent end into the stent lumen for deployment of the Apollo stent to support the proximal end of the Enterprise stent. When navigating the second micro-guidewire into the stent proximal end, try not to rotate the micro-guidewire too much. This technique of double micro-guidewire rescue is also useful for collapsed stents in other curved arteries, especially those with atherosclerotic stenoses that may compress the stent end after withdrawal of the stent delivery system, resulting in collapse of the stent proximal end and decreased blood flow. An arterial curvature may cause the stent proximal segment to form an angle with the curved artery, thus leading to collapse or incomplete opening of the stent proximal end, reduced blood flow, or even complete occlusion of the arterial segment. Elastic retraction of the stenotic vessel may also cause collapse of the stent proximal end if insufficient balloon predilation is performed at the stenotic vascular segment. Insufficient length of the stent may also cause the stent proximal end to collapse because the stent is not long enough to cover the whole stenosis. Rich experience of endovascular maneuver, gentle handling of endovascular devices, sufficient length of the stent deployed, and sufficient balloon predilation of the stenotic vascular segment help reduce intraprocedural complications, especially when withdrawing the stent delivery system. Rude operation may cause an angle formed between the stent and the artery wall, leading to stent collapse and reduced blood flow. Short segments of occlusion are easy to reopen, whereas a long segment of occlusion of a curved artery may readily cause stent deformation and even occlusion, which needs immediate rescuing measures to prevent possible ischemic events.

In endovascular treatment of cerebrovascular diseases, the double micro-guidewire technique is used to increase catheter stability (18), facilitate stent navigation through tortuous arteries (19, 20), and help “Y” configuration stenting in middle cerebral artery bifurcation aneurysms (21) or coil embolization of aneurysms located in the posterior circulation (22). The role of a second micro-guidewire is certainly associated with straightening of the tortuous cerebral artery to facilitate subsequent endovascular maneuver. In our case, the double micro-guidewire technique was used to rescue the collapsed stent proximal end because stent collapse may lead to serious complications of stent occlusion, thrombosis, and ischemic events. The first micro-guidewire passed through the proximal lateral stent mesh into the stent lumen, which not only straightened the tortuous vertebral artery, but also reopened the collapsed stent proximal end. It was, thus, helpful for navigation of the second micro-guidewire right through the opened stent proximal end into the stent lumen for deployment of a supporting stent to prevent collapse of the stent proximal end. When faced with a collapsed stent end in tortuous cerebral arteries, immediate action is needed to rescue the seemingly imminent severe complications of ischemic events. This case report presented an effective approach for solving this severe issue.

## Data Availability Statement

The raw data supporting the conclusions of this article will be made available by the authors, without undue reservation.

## Ethics Statement

The studies involving human participants were reviewed and approved by Ethics Committee of Henan Provincial People's Hospital. The patients/participants provided their written informed consent to participate in this study.

## Author Contributions

KZ and Z-LW: study design. KZ, J-CX, H-LG, B-LG, and Y-FW: data collection. KZ, J-CX, H-LG, and B-LG: data analysis. T-XL: supervision. KZ: writing of the original version. B-LG: revision. All authors contributed to the article and approved the submitted version.

## Conflict of Interest

The authors declare that the research was conducted in the absence of any commercial or financial relationships that could be construed as a potential conflict of interest.

## Publisher's Note

All claims expressed in this article are solely those of the authors and do not necessarily represent those of their affiliated organizations, or those of the publisher, the editors and the reviewers. Any product that may be evaluated in this article, or claim that may be made by its manufacturer, is not guaranteed or endorsed by the publisher.
